# What does the public think about microplastics? Insights from an empirical analysis of mental models elicited through free associations

**DOI:** 10.3389/fpsyg.2022.920454

**Published:** 2022-08-03

**Authors:** Marcos Felipe-Rodriguez, Gisela Böhm, Rouven Doran

**Affiliations:** ^1^Department of Psychosocial Science, University of Bergen, Bergen, Norway; ^2^Department of Psychology, Inland Norway University of Applied Sciences, Lillehammer, Norway

**Keywords:** microplastics, free associations, mental models, personal values, plastic pollution

## Abstract

Microplastics are an issue of rising concern, in terms of their possible implications for both the environment and human health. A survey was distributed among a representative sample of the adult Norwegian population (*N* = 2720) to explore the public understanding of microplastics. Respondents were asked to report the first thing that came to mind when they read or heard the word “microplastics,” based on which a coding scheme was developed that served to categorize the obtained answers into thematic clusters. Results indicate that the public seem to think of microplastics as something bad that might pollute the ocean and harm animal species. Awareness of the sources of microplastics appeared to be rather low, and few respondents mentioned potential ways to solve the problem. Responses differed across certain socio-demographic characteristics; for example, female and younger respondents were more likely to think about the spread and causes/sources of microplastics, whereas a higher educational level was associated positively with thinking of ways to solve the problem. Additional analyses indicated relationships between personal values and the identified thematic clusters; for example, endorsing self-transcendence and openness-to-change values was associated with thinking of ways to solve and of consequences of microplastics. These findings are informative to those wanting to design tailored communications and interventions aimed at reducing plastic pollution and plastic waste.

## Introduction

Public concern about the consequences of microplastics has been growing in recent years, including concern about possible effects on the environment and on human health (European Commission, 2019; SAPEA, 2019). The existing literature suggests that attitudes and knowledge about microplastics can predict various behaviors contributing to the mitigation of related emissions (Deng et al., 2020), and that exploring public opinion and knowledge about plastic litter is pivotal for the successful implementation of policies targeting plastic pollution (Forleo and Romagnoli, 2021). This has led scholars to point out the need to develop insights into public understanding of microplastics in order to develop effective solutions to this evolving global challenge (Heidbreder et al., 2019; Henderson and Green, 2020). The present study takes a mental models approach in order to identify factors that may determine public support for (or opposition to) behaviors and policies addressing microplastics.

Mental models^1^ are mental representations of an event or situation constructed from available information, such as their respective causes and consequences (Bostrom, 2017). There is a growing literature suggesting that mental models can guide policy support and individual behavior in response to environmental challenges. For example, flawed mental models of climate change may be important in explaining support for “wait and see policies” (Sterman and Sweeney, 2007, p. 1). Other studies have employed mental models to reveal subjective beliefs that influence support for when, why, and how species and ecosystems should be conserved (Moon et al., 2019), or to identify factors associated with behaviors among local people from a coastal community that contributes to extensive plastic leakage into the ocean (Phelan et al., 2020). It is commonly assumed that the insights gained from exploring laypeople’s mental models about environmental issues can be used to inform the design of tailored risk communication strategies (Bruine de Bruin and Bostrom, 2013; see also Morgan et al., 2002).

One way of tapping into people’s mental models are free associations, which can be elicited from responses to open-ended questions. This method has been employed to study people’s associations with bioplastics (Dilkes-Hoffman et al., 2019a) but also with plastic more generally (Dilkes-Hoffman et al., 2019b). Few studies have investigated the public understanding of microplastics specifically, and therefore, knowledge about mental models regarding this issue is still scarce. Perhaps the public is not aware of the connections between their personal use of products containing microplastics and environmental pollution, which is important for the consideration of mental models, given the relevance of causes and consequences as their components. An exploration of free associations with microplastics can thus provide novel insights into how laypeople think about the issue, which in turn may predict behaviors and policy endorsements. Associations reflecting causes and consequences might be particularly relevant, since they have been argued to make up the most important components of mental models (Bostrom, 2017).

A lack of public awareness of the issue is illustrated by two empirical studies that conducted focus groups in the United Kingdom. One study investigated perceptions of microplastics in personal care products, concluding that the public might not be aware that consumer goods such as personal care products are sources of microplastics (Anderson et al., 2016). Another study reported that the public, despite frequently associating microplastics with impacts on the ocean, may not understand the process of how microplastics reach marine environments (Henderson and Green, 2020). The picture that emerges from these two studies is that rather than focusing on its origin, associations with microplastics mainly relate to their environmental impacts. It has been argued that these perceptions could be shaped by media narratives that tend to focus on the ubiquity of microplastics and its potential impacts on animal species (Deng et al., 2020; Völker et al., 2020). Media narratives also often highlight the end stages of plastic’s lifecycle (e.g., when plastic can be reused or recycled) instead of focusing on earlier stages (e.g., when plastic gets produced; Dilkes-Hoffman et al., 2019a).

It is well recognized that a person’s value orientation can shape the perceived importance and perceived consequences of environmental issues (Steg et al., 2014). Schwartz (1992, 1994) value theory postulates ten different clusters of basic human values^2^ along two axes; one axis ranging from conservation (security, tradition, and conformity) to openness-to-change (hedonism, self-direction, and stimulation) and the other from self-transcendence (universalism and benevolence) to self-enhancement (hedonism, achievement, and power). Since microplastics can have implications that go beyond concerns for one’s personal interests such as individual health, self-transcendence values might be particularly relevant to understanding people’s associations with microplastics^3^. Other research has demonstrated that a greater emphasis on self-transcendence values is linked to pro-environmental behavior (Liobikienë and Juknys, 2016) and higher concern about plastic litter (Hartley et al., 2018). Thus, those with strong self-transcendence values might be more concerned about potential threats, and more likely to mention consequences, when they think about microplastics. Those who emphasize self-enhancement values might on the other hand be less inclined to consider possible impacts in relation to microplastics, given that self-enhancement values are typically negatively linked with environmental concern (Steg and De Groot, 2012).

### Research aim

This study takes an exploratory approach to provide a better understanding of how the public thinks about microplastics, based on an analysis of responses to an open-ended question. Microplastics are often seen as an issue of public concern due to their potentially harmful consequences for the environment (European Commission, 2019; Deng et al., 2020), and impacts on the environment and the ocean appear to be common associations when people are asked to elaborate on their views on plastic and microplastics (Dilkes-Hoffman et al., 2019b; Henderson and Green, 2020). We therefore expected that consequences, particularly those pertaining to environmental impacts, will be the most prevalent associations in a representative sample of the adult Norwegian population. Additional analyses explored whether individual differences can be predicted by value endorsements, in response to scholars calling for more research on how values may shape public perceptions of microplastics (Pahl and Wyles, 2017; Rist et al., 2018; Kramm et al., 2022).

## Methods

### Sample

We analyzed data that were obtained through the Norwegian Citizen Panel (NCP). The NCP is a research-purpose internet panel based on a probability sample of the general Norwegian population above the age of 18 drawn from the Norwegian National Registry. The NCP runs two to three waves of data collection each year and recruitment is conducted by postal invitation. Participants receive no payment for participating, but in each wave, there is a lottery, where three people win a travel gift card, each valued 8000 NOK. A total of *N* = 2720 respondents were included in the present analysis, which combined data from Wave 11 (2018), Wave 17 (2020), and Wave 18 (2020). Sampling weights for gender, age, education, and geography were applied to compensate for possible sampling bias. The distribution of these four socio-demographic variables in the sample can thus be assumed to reflect that in the general Norwegian population^4^.

### Materials

#### Free associations

Participants responded to the following open-ended question: “What do you think of when you hear or read the word ‘microplastics’?” They received the following instructions: “Please write down the first thing that comes to your mind. We appreciate all kinds of answers, preferably a few sentences, or just a few words if this suits you better.” Similar question wording was employed in previous studies that explored perceptions of plastic-related issues (Dilkes-Hoffman et al., 2019a,b).

A coding system was developed after an initial screening of the responses, alongside consultation of prior research addressing microplastics (e.g., Boucher and Friot, 2017). The resulting coding scheme consists of six superordinate categories, each divided into one or more second- and third-level categories (see Table 1). The complete coding scheme including coding instructions, definitions, and example responses for each category is provided in the Supplementary Material. The six superordinate categories are: (i) ways to solve (i.e., the response indicates that something needs to be done in order for the problem of microplastics to be solved or reduced, or references some type of action to address or tackle microplastics, such as international cooperation or regulations, e.g., “the government needs to penalize plastic usage”), (ii) consequences (i.e., the response refers to potential impacts of microplastics, such as environmental pollution or effects on the economy, e.g., “harming animals”), (iii) evaluations (i.e., the response expresses some type of evaluation of microplastics, such as the importance of the issue or the feasibility or difficulty of tackling the problem, e.g., “they are a very complicated problem to address”), (iv) spread (i.e., the response refers to where microplastics can be found, such as in the ocean, soil or food, e.g., “they are in the air”), (v) sources/causes (i.e., the response refers to where microplastics might originate or come from, or what provokes the release or production of microplastics, such as the plastics industry or washing fleeces, e.g., “car tires”), and (vi) remnant category (i.e., mere descriptions or responses that did not fit with any category).

The responses could be coded at three different levels of specificity. For example, the “consequences” category has three second-level categories (personal, societal, and environmental consequences), which in turn encompass further third-level categories. A response such as “microplastics cause pollution in the environment” would be coded as follows: consequences / environmental consequence / environmental pollution (cf. Table 1). Two university students (native Norwegian speakers) coded the responses after having been trained in using the coding system. First, the two coders coded the responses independently. They agreed on 98.2% of all codes. Then, they were asked to go through the responses they had coded differently and to resolve their disagreements to the extent possible.

**TABLE 1:** Frequencies of free associations with microplastics across each of the identified categories.

Codes			Category	Percentage		
Level 1	Level 2	Level 3		Level 1	Level 2	Level 3
**1**			**Ways to solve**	**10.5**		
	1.1		International level		1.1	
	1.2		National policy level		4.0	
		1.2.1	Regulation *via* incentives			0.6
		1.2.2	Regulation *via* penalties			1.2
		1.2.3	Need for facilitation			1.0
		1.2.4	Increase knowledge			0.6
	1.3		Level of citizens within society		3.0	
		1.3.1	Change behavioral lifestyle			2.0
		1.3.2	Change attitudes and values			0.1
		1.3.3	Collective action			0.3
		1.3.4	Increase awareness			0.8
	1.4		Business and industry		0.5	
	1.5		Respondent engagement		0.8	
		1.5.1	Already taking action			0.4
			Does not want to take action			0.1
**2**			**Consequences**	**51.9**		
	2.1		Personal consequences		5.7	
		2.1.1	Financial resources			0.0
		2.1.2	Personal comfort			0.0
		2.1.3	Personal health			2.4
	2.2		Societal consequences		0.4	
		2.2.1	Societal risks			0.2
		2.2.2	Social justice / equity			0.0
		2.2.3	Economy			0.0
	2.3		Environmental consequences		47.9	
		2.3.1	Environmental pollution			21.5
		2.3.2	Environmental preservation			0.0
		2.3.3	Environmental aesthetics			0.0
		2.3.4	Consequences for animals			10.6
		2.3.5	Consequences for plants			0.5
		2.3.6	Consequences for the food chain			3.8
**3**			**Evaluations**	**36.5**		
	3.1		Concerning feasibility to tackle		10.7	
		3.1.1	Easy to tackle			0.1
		3.1.2	Difficult to tackle			10.6
	3.2		Concerning effectiveness of potential measures		0.8	
	3.3		Concerning importance		2.5	
		3.3.1	Important for the present			0.4
		3.3.2	Important for the future			0.9
	3.4		Expressions of skepticism		1.1	
		3.4.1	Skepticism toward underlying intentions of stakeholders			0.4
		3.4.2	Skepticism toward scientific understanding			0.1
	3.5		Expressions of affective valence		27.6	
		3.5.1	Positive valence			0.7
		3.5.2	Negative valence			26.5
	3.6		Expressions of conflict-laden aspects		2.0	
		3.6.1	Conflict between different impacts			1.1
		3.6.2	Conflict between different generations			0.5
**4**			**Spread**	**48.7**		
	4.1		Aquatic environments		34.1	
		4.1.1	Saltwater			31.6
		4.1.2	Rivers			0.8
		4.1.3	Lakes			0.3
	4.2		Land / soil		2.1	
	4.3		Air		0.3	
	4.4		Animals		8.9	
		4.4.1	Fish			5.7
		4.4.2	Whales			1.4
	4.5		Plants/flora		0.3	
	4.6		Drinking water		1.3	
	4.7		Food		4.5	
	4.8		Humans		5.2	
**5**			**Causes/sources**	**24.1**		
	5.1		Fleece, clothing		9.4	
	5.2		Sewage treatment		0.2	
	5.3		Car tires		0.7	
	5.4		Artificial grass turf		3.3	
	5.5		Litter		7.4	
	5.6		Personal care products		3.2	
	5.7		Agriculture		0.1	
	5.8		Paint		0.1	
	5.9		Industry		2.3	
		5.9.1	Fishing		0.4	
		5.9.2	Aquaculture		0.1	
**6**			**Remnant**	**12.3**		
	6.1		Mere descriptions		6.8	
	6.2		Non-codable responses		4.1	
	6.3		Does not know		1.4	

Percentages are based on *n* = 2527 (adjusted for weights). First-level categories are displayed in bold. Sampling weights for gender, age, education, and geography were applied in the analyses.

#### Personal values

Personal values were measured *via* the Ten-item Value Inventory (TIVI; Sandy et al., 2017), an ultra-brief version of the Portrait Values Questionnaire (PVQ; Schwartz, 2003). The 10 values assessed by the TIVI are as follows: conformity, tradition, benevolence, universalism, self-direction, stimulation, hedonism, achievement, power, and security. The order of the items was randomized per respondent. Universalism, benevolence, achievement (reversed) and power (reversed) values were combined into the self-transcendence versus self-enhancement dimension. Conformity, security, stimulation (reversed), self-direction (reversed) and hedonism (reversed) values were combined into the conservation versus openness-to-change dimension. Higher positive values represent more self-transcendence and more conservation values, relative to self-enhancement and openness-to-change, respectively; for a similar approach studying the relative importance of personal values for explaining public perceptions of environmental issues, see Poortinga et al. (2019). We treated each individual’s mean response to all items as a covariate to partial out the effect of individual differences in mean response level, while leaving the distribution of responses within individuals unchanged (Schwartz, 1992).

#### Socio-demographics

In addition to gender (1 = male, 2 = female) and age (1 = born 1959 or earlier, 2 = born 1960-1989, 3 = born 1990 or later), the analyses incorporated a categorical measure on education (1 = completed primary school or below, 2 = completed secondary school, 3 = college or university degree). These socio-demographics were included as covariates based on previous studies addressing public perceptions of plastic-related issues; for example, women tend to report greater concern about plastic pollution (Dilkes-Hoffman et al., 2019b; Forleo and Romagnoli, 2021) and stronger behavioral intentions to engage in mitigation actions (Hartley et al., 2018; Deng et al., 2020).

### Analyses

First, we inspected the frequencies of the categories in the coding scheme, excluding the remnant category. To assess if the obtained responses reflect different degrees of richness, we calculated the average word count for each response. An individual respondent’s word count was considered as a dimension of richness (cf. Andrews and Lamb, 2017). To explore whether thinking about some topics co-occurs with thinking about other topics, we calculated correlations among the main categories. Second, we conducted multiple logistic regression analyses to assess how well socio-demographics and personal values predict free associations with microplastics, with the main categories as the criterion. All analyses were carried out with IBM SPSS Statistics for Windows, Version 27.

## Results

### Free associations

**FIGURE 1:**
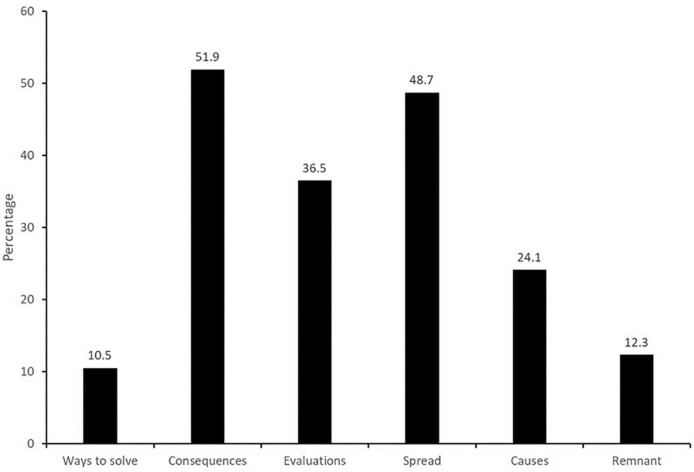
Frequencies of first-level categories (percentages).

Figure 1 shows that consequences of microplastics (51.9%) were the most frequent main category, dominated by references to environmental impacts (cf. Table 1). Only a few responses referenced personal consequences, and almost no references to societal consequences were made. Some of the mentioned environmental consequences could further be specified into environmental pollution, followed by impacts on animals and consequences for the food chain.

The second most frequent category was spread (48.7%), which indicates where respondents believe that microplastics can be found. Within this category, references to aquatic environments dominated the responses, comprising saltwater, rivers and lakes. The next most frequent second-level category is spread of microplastics to animals, among which most respondents mentioned fish and only a few mentioned whales. Second-level categories within spread, that were comparable in terms of frequency, included humans, food, and soil. The least frequent second-level categories regarding where microplastics can be found were drinking water, plants and the air.

The third most frequent main category by a notable margin was evaluations (36.5%). The most prevalent type of evaluation regarding microplastics were expressions of affective valence, which could be positive or (predominantly) negative. The second most frequent type of evaluation was evaluations concerning the feasibility of addressing microplastics, which were dominated by views that microplastics are a difficult problem to tackle. Few responses reflected evaluations concerning the importance of microplastics, conflict-laden aspects of microplastics, skepticism, or statements regarding the effectiveness of potential solutions.

Sources/causes of microplastics, that is, references to where microplastics are believed to come from or be produced, were mentioned in approximately a quarter of the total responses (24.1%). The most frequent second-level categories among potential sources of microplastics were fleece and clothing and litter. A few responses mentioned artificial grass turf, personal care products, and industry, while a very small number mentioned car tires, sewage treatment, agriculture and paint.

The least frequent of the main categories was ways to solve the problem (10.5%). References to actions that might or should contribute to solving or mitigating the problem were dominated by responses referring to national policies, among which penalties were the most frequent third-level category. Slightly fewer responses mentioned demands of citizens within society, and even fewer referred to the international level.

**FIGURE 2:**
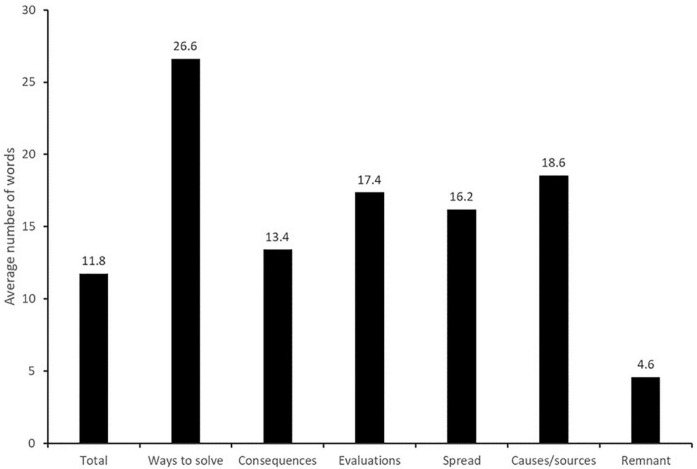
Average number of words of responses for each first-level category.

Figure 2 shows that average word count per response was 11.8. The category with the highest average word count by far is ways to solve the problem of microplastics (26.6), followed by causes/sources (18.6), evaluations (17.4), spread (where microplastics can be found; 16.2) and consequences (13.4). The remnant category has by far the lowest average length (4.6).

The category showing the highest correlations with other categories was evaluations. Table 2 shows that expressing an evaluation correlated positively with ways to solve and with consequences, but negatively, and less strongly, with sources/causes and with spread. Moreover, a small negative correlation was found between consequences and causes/sources, whereas a small positive correlation was observed between spread and causes/sources. Lastly, ways to solve showed small positive correlations with causes/sources and with consequences, and a small negative correlation with spread.

**TABLE 2:** Intercorrelations among free associations with microplastics.

Variables	1	2	3	4	5
1. Ways to solve	-				
2. Consequences	0.044**	-			
3. Evaluations	0.240***	0.319***	-		
4. Spread	−0.060**	0.007	−0.105***	-	
5. Causes/sources	0.057**	−0.099**	−0.081***	0.117***	-

Pearson correlations (two-tailed). **p < 0.01 and ***p < 0.001.

### Predicting free associations from socio-demographics and personal values

We conducted a series of multiple logistic regression analyses, each considering one of the main categories of free associations with microplastics as the dependent variable and socio-demographics and personal value orientations as predictors (see Table 3). While an odds ratio greater than 1 indicates a positive association (e.g., with endorsing self-transcendence values), an odds ratio lower than 1 indicates a negative association (e.g., with endorsing conservation values). Gender was entered as a dichotomous predictor, with male respondents as the reference category; the corresponding odds ratio represents the likelihood for females to mention a main category compared to the likelihood for males. Age was entered as a continuous predictor, with three groups ordered from youngest to oldest; the odds ratios represent the likelihood that someone older thinks of ways to solve microplastics, for example, compared to the likelihood for younger respondents. Education level was treated as a continuous predictor, ranging from the lowest to highest level of education; odds ratios higher than 1 indicate that a higher educational level increases the odds of the corresponding outcome.

**TABLE 3:** Logistic regressions predicting free associations with microplastics.

	Ways to solve		Consequences		Evaluations		Spread		Causes/sources	
Variables	OR	95% CI	OR	95% CI	OR	95% CI	OR	95% CI	OR	95% CI
**Socio-demographics**										
Gender (Female)	0.674	[0.510, 0.890]	1.115	[0.941, 1.322]	1.212	[1.016, 1.446]	1.239	[1.044, 1.470]	1.664	[1.361, 2.034]
Age group	1.140	[0.930, 1.397]	0.909	[0.802, 1.030]	1.095	[0.962, 1.247]	0.782	[0.690, 0.887]	0.794	[0.690, 0.959]
Education	1.010	[1.002, 1.018]	0.994	[0.987, 1.000]	1.006	[1.000, 1.013]	1.005	[0.999, 1.012]	1.005	[0.998, 1.012]
**Personal values**										
Self-transcendence vs. self-enhancement	1.250	[1.004, 1.557]	1.191	[1.041, 1.364]	1.026	[0.982, 1.181]	0.938	[0.820, 1.074]	1.145	[0.977, 1.342]
Conservation vs. openness-to-change	0.723	[0.575, 0.908]	0.831	[0.722, 0.957]	0.950	[0.821, 1.100]	0.987	[0.779, 1.033]	0.81	[0.689, 0.959]
Constant	0.082**		0.628		0.203**		1.629		0.588	
Model χ^2^	25.391***		25.986***		17.526**		39.500***		49.153***	
Nagelkerte (pseudo R^2^)	0.023		0.015		0.011		0.023		0.032	

CI, confidence interval for odds ratio (OR).

Sampling weights for gender, age, education, and geography were applied in the analyses. **p < 0.01 and ***p < 0.001.

Males are more likely than females to think about ways to solve the problem of microplastics. A higher level of education increases the odds of mentioning ways to solve microplastics. Furthermore, both value dimensions are significantly related to thinking about ways to solve the problem. Those who prioritize self-transcendence over self-enhancement have higher odds of mentioning ways to solve microplastics, whereas those who prioritize conservation over openness-to-change have lower odds of thinking of potential ways to solve the problem of microplastics.

Moreover, both value dimensions were significantly related to referencing consequences of microplastics. While those prioritizing self-transcendence values over self-enhancing values have higher odds of thinking about consequences, those prioritizing conservation values over openness-to-change values have lower odds of referencing consequences when thinking about microplastics.

Concerning evaluations of microplastics, females are more likely to give evaluations than males. Females also have higher odds than males of mentioning the spread of microplastics (i.e., where microplastics can be found). People higher in age have lower odds of referencing the spread as well as the causes/sources of microplastics.

Lastly, females have higher odds than males of thinking about where microplastics come from or are produced. When it comes to personal values, those who endorse conservation values have lower odds of thinking about sources of microplastics. Predictors not mentioned in the preceding paragraphs did not exhibit a significant association with the corresponding outcome category.

## Discussion

Respondents mainly associated microplastics with possible consequences, often in connection with environmental consequences, and less often in relation to personal impacts. While it was also mentioned where microplastics can be found, such as in aquatic environments and the ocean, expressed views on possible causes/sources appeared to be somewhat vague. This is in line with studies suggesting that the general public might not be very aware of the sources of microplastics (Anderson et al., 2016; Deng et al., 2020; Henderson and Green, 2020). Clothing and litter were the most common sources people thought of, and there were only very rare references to other relevant sources of microplastics such as car tires or industry. One interpretation is that people do not seem to fully understand the processes by which microplastics end up in the ocean, as already noted in the existing literature (Henderson and Green, 2020).

Ways to solve the problem of microplastics made up the least frequent association, and if this category was mentioned at all, the obtained answers were very unspecific. Similar findings have been reported in other studies (e.g., Anderson et al., 2016; Henderson and Green, 2020). Although evaluations were a frequent association people made in our study, the types of evaluations people made mostly concerned ascribing a negative affective valence to microplastics, and, to a lesser extent, reflecting on the feasibility of tackling the problem. The finding that most respondents did not mention and may not be aware of potential ways to solve the problem might contribute to the lack of more varied types of evaluations. It is possible that if participants were more aware of possible ways to reduce microplastics pollution, they would make more varied evaluations concerning different aspects of microplastics, such as the importance of the issue or the effectiveness of these potential solutions. This complements existing literature calling for greater communication efforts in order to focus on solutions as well as threats concerning microplastics (Veiga et al., 2016).

A large proportion of our respondents associated microplastics with something bad, which might reflect patterns in how the media report on the topic. Völker et al. (2020) conducted an empirical analysis of media framings and argue that media reports use three main narratives: (i) that microplastics are present in the environment in large numbers, (ii) that microplastics are present in food and beverages, and (iii) that microplastics contain toxic chemicals which might be ingested by animals. Our results showed that many respondents indeed associated microplastics with their presence in the environment, mostly marine environments, but also perceived a connection with environmental pollution and harming animals. The narrative of finding microplastics in food and beverages did not resonate within the surveyed population, with only a few respondents making these associations (for similar findings, see Henderson and Green, 2020).

One aspect contributing to the observed pattern of associations might be the tendency to relate plastic to stages at the end of its lifecycle, such as the moment of purchase or the moment of release into the natural environment, rather than to its production and other characteristics. This tendency could be due to the end-of-life being the stage at which consumers interact with plastic packaging, making them feel responsible for decisions regarding its disposal (Herbes et al., 2018), or that end-of-life impacts are easier to understand and communicate, meaning that this stage is most often discussed in the media (Dilkes-Hoffman et al., 2019a). Our analyses regarding the richness of responses seems to support this interpretation: references about ways to solve the problem were on average the longest, whereas references to consequences were the shortest. It has been argued that message length is an indicator for deliberation in online communications (Liu and Zhang, 2020). Therefore, the differences in richness between consequences and the other categories, together with the fact that references to consequences were generally most prevalent, could mean that laypeople associate microplastics with their consequences more intuitively than with possible sources or with ways to solve the problem.

Female respondents were more likely to think of where microplastics are found as well as of their sources/causes. Nonetheless, they were less likely to think of ways to solve the problem. Younger respondents thought more frequently of the spread and sources of microplastics. This is in line with findings that age can predict attitudes and intentions in related domains, such as marine threats (Lotze et al., 2018), beach litter (Rayon-Viña et al., 2018), and concern over the health of marine environments (Potts et al., 2016). People with higher education levels were more likely to think of ways to solve the problem of microplastics, aligning with studies investigating individual differences in perceptions of plastic pollution more broadly (Hartley et al., 2018; Deng et al., 2020; but see Dilkes-Hoffman et al., 2019b). Education has been argued to play a key role in increasing awareness and changing behaviors and attitudes to preserve environmental resources (Forleo et al., 2019) and education campaigns are considered necessary for the implementation of successful policies to mitigate plastic marine pollution (Clayton et al., 2021).

Aside from demonstrating associations with socio-demographics, the analyses suggest that perceptions about microplastics can (to some extent) be predicted from a person’s value orientation. This was indicated by the finding that endorsing self-transcendence (rather than self-enhancement) values was associated with considering potential consequences of microplastics, and with a greater likelihood to think of potential ways to solve the problem. Individual endorsements of conservation (rather than openness-to-change) values was negatively related to associating microplastics with ways to solve, consequences, and causes/sources. These findings corroborate studies that have linked self-transcendence values with concern about potential threats posed by plastic litter in the more general sense (Hartley et al., 2018), and expand upon literature that has reported mixed findings regarding the influence of conservation and openness-to-change values on environmental perceptions (e.g., Schultz and Zelezny, 1999; Milfont et al., 2015; Poortinga et al., 2019).

## Limitations

Free associations are a non-directive method of eliciting spontaneous connections that people make when they are asked to think about a word or expression (Dany et al., 2015). While this method can be useful in particular when there is not much knowledge about a given topic, its application in research addressing the public understanding of microplastics remains not without certain challenges. One such challenge concerns the notion that categorizing content into thematic groupings (or clusters) may not be independent from the meaning that the investigating researchers ascribe to the obtained responses (Lo Monaco et al., 2017). We aimed to account for this by employing two independent coders so that interpretations would not be made by a single person, and by developing a multilevel coding scheme so that each answer could be categorized at different levels of specificity. An alternative way to improving reliability of thematic groupings might have been to ask respondents to express the meaning that they would like to give to their association (e.g., Piermattéo et al., 2014), and/or to ask them for a justification for all associated terms (e.g., Galli and Fasanelli, 2020).

## Conclusion

The present study provides incremental evidence about what members of the public associate with microplastics, with references to consequences and the spread of microplastics making up the most common themes among the surveyed population. A substantial share believed that microplastics can accumulate in marine environments, produce environmental pollution, have negative impacts on animal species, and on a more general note, they perceived microplastics as something bad and harmful. Rather than focusing predominantly on potential harmful impacts, information campaigns may benefit from combining facts about specific sources of microplastics with practical guidance on how individual actions in everyday life might contribute to mitigating the problem. This follows the notion that knowledge of behavioral options and potential action strategies are among the most important types of knowledge associated with pro-environmental behavior (e.g., Kollmuss and Agyeman, 2002). Future research could aid the development of such campaigns by focusing on more concrete aspects related to microplastics, and by considering further personal and structural factors that may shape how members of the public perceive the risks and benefits of microplastics.

## Data availability statement

The raw data supporting the conclusions of this article will be made available by the authors, without undue reservation.

## Ethics statement

Ethical review and approval was not required for this study in accordance with the local legislation and institutional requirements. The Norwegian Citizen Panel dataset, on which this study is based, deals with human subjects and follows the EU General Data Protection Regulation (GDPR). According to this, a Data Protection Impact Assessment (DPIA) was conducted and approved by the University of Bergen. The DPIA was conducted in cooperation with the Norwegian Agency for Shared Services in Education and Research (Sikt). The DPIA number is 118868. In addition, the Scientific Committee of the Norwegian Citizen Panel reviews all questions that are to be fielded in each panel wave. Participation in the panel is by written informed consent, and data are always treated confidentially.

## Author contributions

MFR, GB, and RD contributed to the conception and design of the study. MFR performed the statistical analyses and wrote the first draft of the manuscript. All authors contributed to manuscript revision, and read and approved the final manuscript.

## Footnotes

1 This conceptualization of mental models shares conceptual similarities with social representations, which have been defined as “organized sets of opinions, knowledge, attitudes, and beliefs about a social object … co-constructed and shared by a social group” (Fasanelli et al., 2020, p. 1).

2 These are “desirable trans-situational goals, varying in importance, that serve as guiding principles in the life of a person or other social entity” (Schwartz, 1994, p. 21).

3 Self-transcendence values are those which go beyond a focus on the self and prioritize the welfare of other people, the community, or the environment (Cheung et al., 2014). In contrast, self-enhancement values give little consideration to others in personal decision-making and lead to attitudes and behaviors focused on the self (Urien and Kilbourne, 2011).

4 For details, including recruitment and sampling procedures for each panel wave, see https://nsd.no/nsddata/serier/norsk_medborgerpanel.html.
