# Supplementary material for: What does the public think about microplastics? Insights from an empirical analysis of mental models elicited through free associations
Source: Front Psychol. 2022 Aug 3;13:920454. doi: 10.3389/fpsyg.2022.920454 (PMC9384851; doi:10.3389/fpsyg.2022.920454)
Supplement: Supplementary file 1 [file Data_Sheet_1.DOCX]

**Instructions for coders and description of categories**

**Marcos Felipe Rodriguez^*^, Gisela Böhm, Rouven Doran**

***correspondence:** [marcos.felipe.rodriguez@uib.no](mailto:marcos.felipe.rodriguez@uib.no)

**Coding Instructions for Free Associations**

We ask for your help in categorizing responses that were given by participants in a study on perceptions on microplastics. The aim of the study is to find out what people think about microplastics (i.e., small plastic particles produced as such and also originated from the degradation of larger plastic objects). We presented the following open-ended question to participants:

**Please describe briefly the first thing that comes to mind when you hear or read the word “microplastics”**

Participants were instructed that they could either respond with one word, a few words, or complete sentences and paragraphs.

We want you to categorize the responses to this question according to a coding scheme, which you find below.

**Coding Scheme**

We provide you with a detailed coding system, which we ask you to use in order to categorize a response. You will see that the responses differ a lot in specificity. For example, some people just say “small plastic” very generally; others are more specific and say something like “pollution in the sea and rivers, they come from washing of clothes”. Accordingly, the categories in our coding system differ in their specificity in order to capture these differences. That is, the categories have subcategories.

In the table below you find for each category a description, 1-3 examples of responses that would fall into this category, and a code (a number).

Study this table carefully.

When coding the responses, use your best judgment as to what the respondent meant when giving the response. Responses might belong to more than one category.

The coding scheme has three levels of specificity. The code for a category indicates the level of specificity, the more digits a code has the more specific it is. For example, code 11 is a subcategory of code 1; code 111 is a subcategory of code 11. Always use the most specific category possible. Use a superordinate category if the response does not give more specific information to assign it to a subcategory or if something specific is said which does not match any of the subcategories.

| **Code** | | | | **Column** | | **Category** | **Description** | **Example(s)** |
| --- | --- | --- | --- | --- | --- | --- | --- | --- |
| **Level 1** | | **Level  2** | **Level 3** |  | |  |  |  |
| 1 | |  | | C | | Ways to solve | The response indicates that something is needed for the problem to be reduced/solved.  No further specification on what would contribute to its reduction/solution | - *Noe må gjøres* |
|  | | 11 |  | D | | International level | The response indicates that some action on an international or global level is required. | - *Må løses internasjonalt* |
|  | | 12 |  | E | | Level of national policies | The response indicates that some political action or policy on the national level is required.  It is not specified what kind of political action or policy is needed. Or what is said does not match subcategories 121 to 124. | - *Våre politikere må gjøre mer* |
|  | |  | 121 | F | | Regulation via incentives | The response indicates that the government (nation) should introduce regulations that reward certain actions. | - *Flere vil resirkulere plast hvis de til og med får litt penger for det* |
|  | |  | 122 | G | | Regulation via punishments | The response indicates that the government (nation) should introduce regulations that punish certain actions. | - *Forbruk av mikroplast kan reduseres ved å ha høyere avgifter på produkter som inneholder det* |
|  | |  | 123 | H | | Need for facilitation (availability) | The response indicates that structural changes are needed to promote behaviour change. | - *Flere ville ha brukt alternative materialer til plast hvis det var mer tilgjengelig, f.eks. i butikker* |
|  | |  | 124 | I | | Need to increase knowledge (general public and research) | The response indicates that more information is required, both in science and among the public. | - *Vi trenger mer forskning på dette temaet* - *Offentligheten trenger mer informasjon* |
|  | | 13 |  | J | | Requirement on the level of the citizens within a society | The response indicates that some change is required concerning the citizens in a society.  It is not specified what kind of change is needed. Or what is said does not match subcategories 131 to 134. | - *Folk må forandre seg* - *Vi må gjøre noe* |
|  | |  | 131 | K | | (Need to) change behaviour/lifestyles | The response indicates that there is a need for changes in behaviours and/or lifestyles. | - *Folk må endre atferden sin* - *Folk bør bruke færre plastposer* |
|  | |  | 132 | L | | (Need to) change attitudes/values | The response indicates that there is a need for changes in attitudes and/or values. | - *Folk må endre sine holdninger / hva de tenker* |
|  | |  | 133 | M | | Need for collective action | The response indicates that there is a need for collective action. | - *Alle må være med* |
|  | |  | 134 | N | | Need to increase engagement/awareness | The response indicates that people need to become more involved about the issue. | - *Vi trenger økt bevissthet om vårt eget forbruk av produkter som inneholder mikroplast* |
|  | | 14 |  | O | | Way to solve in the level of business and industry | The response mentions that businesses/industry need to change to address the problem effectively | - *Plastindustrien må endre seg* |
|  | | 15 |  | P | | Engagement | The response indicates that they want to take action | - *Jeg vet ikke hvordan jeg kan hjelpe* |
|  | |  | 151 | Q | | Respondent already taking action | The response indicates that they are already addressing the problem | - *Jeg resirkulerer på den beste måten jeg kan* |
|  | |  | 152 | R | | Respondent does not want to take action | The response indicates that they are not currently willing to address MP | - *Jeg gidder ikke å resirkulere* |
| 2 | |  | | S | | Consequences | The response refers to potential consequences. These can be positive or negative.  It is not specified what kind of consequence or who (or what) would be affected. Or what is said does not match subcategories 21 to 23. | - *Det har dårlige konsekvenser* |
|  | | 21 |  | T | | Personal consequences | The response refers to consequences of MP that affect the respondent him-/herself or people in general. The consequences can be positive or negative.  The consequences are not further specified. Or what is said does not match subcategories 211 to 216. | - *Det ville ha negative konsekvenser for meg* - *Det ville være til fordel for meg.* - *Det ville ha ulemper for folk* - *Det ville være bra for folk* |
|  | |  | 211 | U | | Personal financial resources | The response indicates that MP affect or might affect the financial resources that the respondent/people have at their disposal. | - *Mikroplast vil påvirke fiskernes profitt* |
|  | |  | 212 | V | | Personal comfort | The response indicates that MP affect personal comfort. | - *Mikroplast er ubehagelig for meg/folk* |
|  | |  | 213 | W | | Personal health effects | The response indicates that personal health is affected. | - *Mikroplast kan påvirke folks helse* |
|  | | 22 |  | X | | Societal consequences | The response refers to consequences of the step that affect society. The consequences can be positive or negative.  The kind of societal consequence is not further specified. Or what is said does not match subcategories 221 to 223. | - *Mikroplast påvirker samfunnet vårt* |
|  | |  | 221 | Y | | Societal risks | The response indicates that the MP pose a risk for society. | - *Mikroplast har negativ innvirkning på hvordan folk lever sammen i lokalsamfunn* - *Mikroplast er farlig for samfunnet* |
|  | |  | 222 | Z | | Social justice/equity | The response indicates that MP can be fair, or it can lead to differences in opportunities/influence. | - *Mikroplast påvirker folk med mindre penger, men ikke de rike* |
|  | |  | 223 | AA | | Economy | The response indicates that MP affect or could affect the economy generally, not just to a few people | - *Mikroplast vil ha innvirkning på økonomien vår* |
|  | | 23 |  | AB | | Environmental consequences | The response refers to consequences of the step for the natural environment. The consequences can be positive or negative.  The kind of environmental consequence is not further specified. Or what is said does not match subcategories 231 to 233. | - *Det vil påvirke miljøet* |
|  | |  | 231 | AC | | Environmental pollution | The response indicates that MP will affect the degree of pollution. | - *Mikroplast forårsaker forurensning* |
|  | |  | 232 | AD | | Environmental preservation | The response indicates that MP contribute/will contribute to preserving the environment. | - *Mikroplast er bra for miljøet* |
|  | |  | 233 | AE | | Environmental aesthetics | The response indicates that MP has implications for how the environment looks. | - *Mikroplast får havet til å se stygt ut* |
|  | |  | 234 | AF | | Consequences on animals | When it is mentioned that MP might have a specific impact on animals | - *Mikroplast er skadelig for dyr* |
|  | |  | 235 | AG | | Consequences on plants | It is indicated that MP might have a specific impact on | - *Mikroplast er skadelig for planter* |
|  | |  | 236 | AH | | Consequences on the food chain | It is indicated that MP might affect the food chain | - *Mikroplast påvirker næringskjeden* |
| 3 | |  | | AI | | Evaluation | The respondent expresses an evaluation, but does not clearly indicate to which aspect of MP he/she refers.  Or what is said does not match subcategories 31 to 36. | - *Utmerket* - *Interessant* |
|  | | 31 |  | AJ | | Evaluation concerning feasibility to reduce/tackle MP | The response refers to how easy/hard it is to tackle/solve the problem of MP | - *Jeg er usikker på hvor gjennomførbart det er å håndtere mikroplast* |
|  |  |  | 311 | AK | | Easy to tackle | The response indicates that solving/reducing MP is or would be easy | - *Det er enkelt å redusere mikroplast* |
|  |  |  | 312 | AL | | Difficult to tackle | The response indicates that solving/reducing MP is or would be difficult | - *Mikroplast er et vanskelig problem å løse* |
|  | 32 | |  | AM | | Evaluation concerning effectiveness of potential solutions or measures | The response indicates that something is (or isn’t) effective to reduce/tackle MP (or is not) effective. | - *Dette har liten effekt* - *Dette er som en dråpe i havet* |
|  | | 33 |  | AN | | Evaluation concerning importance | The response indicates that MP are or are not important.  It is not specified in which sense MP (or are not) important. Or what is said does not match subcategories 331 or 332. | - *Mikroplast betyr mye* |
|  | |  | 331 | AO | | Importance for the present | The response indicates that MP are important today. | - *Det er viktig at vi håndterer dette i dag* - *Det haster med å redusere mikroplast* |
|  | |  | 332 | AP | | Importance for the future | The response indicates that MP will be important in the future. | - *Mikroplast vil være et viktig problem i fremtiden* |
|  | | 34 |  | AQ | | Expression of scepticism | The respondent expresses general scepticism, without further specification. Or what is said does not match subcategories 341 or 342. | - *Jeg tror ikke på det* |
|  | |  | 341 | AR | | Scepticism towards underlying intentions of stakeholders | The response implies suspiciousness that producers, manufacturers, governments, etc. might not want to address the problem | - *Regjeringen ønsker ikke å fokusere på mikroplast fordi det ikke lønner seg* |
|  | |  | 342 | AS | | Scepticism towards the scientific understanding | The response implies suspiciousness and scepticism towards the scientific understanding of MP | - *Mikroplast er faktisk ikke godt forstått av vitenskapen* |
|  | | 35 |  | AT | | Expression of affective valence | The respondent expresses an affective evaluation. It is not clear whether the evaluation is positive or negative. | - *Jeg har ikke sterke følelser om mikroplast* |
|  | |  | 351 | AU | | Positive affect | The respondent expresses positive affect. | - *Bra* - *Flott* |
|  | |  | 352 | AV | | Negative affect | The respondent expresses negative affect. | - *Dårlig* - *Skummelt og farlig* |
|  | | 36 |  | AW | | Expression of conflicting aspects | The response indicates that MP have different aspects that conflict (or contradict each other).  The type of conflict is not specified. Or what is said does not match subcategories 361 to 362. | - *Mikroplast har positive og negative sider* |
|  | |  | 361 | AX | | Conflict between different impacts | The respondent links both positive and negative factors with MP. | - *Dårlig for miljøet, men billig* |
|  | |  | 362 | AY | | Conflict between different generations | The response implies differences in how one generation acts, or will have to act, compared to another generation. | - *Fremtidige generasjoner må betale prisen for dette* |
| 4 | |  | | AZ | | Spread | The response refers to the spread of MP, where can they be found, where they can reach out to  No further specification concerning the context of the spread is given. Or what is said does not match 41- | - *Det er overalt* |
|  | | 41 |  | BA | | Aquatic environments | The response indicates that MP reach out to water environments | - *Det kommer inn i vannet* |
|  | |  | 411 | BB | | Salt-waters | The response indicates that MP get to the ocean | - *Det finnes i sjøen* |
|  | |  | 412 | BC | | Rivers | The response indicates that MP get to rivers | - *Det finnes i elver* |
|  | |  | 413 | BD | | Lakes | The response indicates that MP get to lakes | - *Det finnes i innsjøer* |
|  | | 42 |  | BE | | Land/soil | The response indicates that MP get in the soil | - *Det finnes i jorden* |
|  | | 43 |  | BF | | Air | The response indicates that MP are present in the air | - *Det er i lufta* |
|  | | 44 |  | BG | | Animals | The response indicates that MP get into animals | - *Det kommer seg inn i dyr* |
|  | |  | 442 | BH | | fish | The response indicates that MP get into fish | - *Det kommer seg inn i fisk* |
|  | |  | 443 | BI | | whale | The response indicates that MP get into whales | - *Det kommer seg inn i en hval* |
|  | | 45 |  | BJ | | Plants/flora | The response indicates that MP get into plants | - *Det kommer seg inn i planter* |
|  | | 46 |  | BK | | Drinking water | The response indicates that MP can get in the water we drink | - *Det er i vårt drikkevann* |
|  | | 47 |  | BL | | Food | The response indicates that MP can get in our food | - *Det kan være mikroplast i mat* |
|  | | 48 |  | BM | | Humans | The response indicates that MP can be found in humans | - *Det er til og med funnet i menneskekropper* |
| 5 | |  |  | BN | | Causes/sources | The response indicates what causes MP to be released or where they come from  It is not clear what source is being referred to, or it does not match 51-58 | - *Mikroplast har ulike kilder* |
|  | | 51 |  | BO | | Fleece/clothing | The response indicates that MP might come from fleece or clothing | - *Fra vasking av klær* |
|  | | 52 |  | BP | | Sewage treatment | The response | - *Kloakkrenseanlegg renser ikke vannet helt* |
|  | | 53 |  | BQ | | Car/truck tires | The response | - *Bildekk produserer det* |
|  | | 54 |  | BR | | Artificial grass turfs | The response | - *Fra kunstgressbaner* |
|  | | 55 |  | BS | | Litter | The response indicates that a source of MP might be littering or waste | - *Kommer fra akkumulert forsøpling* |
|  | | 56 |  | BT | | Personal care products | The response | - *Fra kosmetikk* |
|  | | 57 |  | BU | | Agriculture | The response | - *Fra produkter som brukes i landbruk* |
|  | | 58 |  | BV | | Paint | The response indicates that MP might come from paint | - *Fra båtmaling* |
|  | | 59 |  | BW | | Industry | The response indicates that a source of MP has to do with an industry, not specified which | - *Fra industri som ikke håndterer plast på en god måte* |
|  | |  | 591 | BX | | Fishing | The response indicates that the fishing industry is a source of MP | - *Fra fisking* |
|  | |  | 592 | BY | | Aquaculture | The response indicates that aquaculture is a source of MP | - *Fra akvakulturer* |
|  | | | | | **Remnant categories** | | | |
|  | | 61 |  | CA | | Mere description | The respondent gives a mere description or rephrasing of MP | - *Liten plast* |
|  | | 62 |  | CB | | Non-codeable response | The response does not match any of the categories. |  |
|  | | 63 |  | CC | | Don’t know | The respondent indicates that he/she does not know an answer. | - *Vet ikke* - *Har ikke hørt om det* |
